# Social Determinants of Cardiovascular Health: Yes, They Matter—But What Can We Do to Address Them?

**DOI:** 10.1007/s11886-025-02336-2

**Published:** 2026-02-23

**Authors:** Faith E. Metlock, Mathias Lalika, Joshua J. Joseph, Yvonne Commodore-Mensah, LaPrincess C. Brewer

**Affiliations:** 1 Johns Hopkins School of Nursing, Baltimore, MD USA; 2 Department of Cardiovascular Medicine, Mayo Clinic College of Medicine, 200 First St. SW, Rochester, MN 55905 USA; 3 Center for Clinical and Translational Science, Mayo Clinic, Rochester, MN USA; 4 The Ohio State University Wexner Medical Center, Columbus, OH USA; 5 Clinical and Translational Science Institute, The Ohio State University, Columbus, OH USA

**Keywords:** Social determinants of health, Social drivers, Cardiovascular health, Interventions

## Abstract

**Purpose of Review:**

Awareness of how social determinants of health (SDoH) shape cardiovascular outcomes is increasing, yet evidence on effective interventions remains limited. This review examines the associations between Healthy People 2030 SDoH domains and cardiovascular health (CVH), defined by the American Heart Association’s Life’s Essential 8 (LE8), and highlights interventions with potential to reduce disparities.

**Recent Findings:**

Adverse SDoH—including economic instability, limited education and healthcare access, neighborhood disadvantage, and low social support—are consistently linked to lower LE8 scores and higher CVH burden, especially in under-resourced communities. Promising interventions include produce prescriptions, culturally tailored education, community health worker integration, built environment enhancements, and peer support models. However, most studies target single determinants, involve short follow-up, or focus on limited populations, leaving gaps in scalability and equity impact.

**Summary:**

Addressing SDoH is essential for improving LE8 behaviors and factors. Progress requires multilevel, equity-centered strategies that align clinical care, policy, and research, while expanding rigorous trials to guide sustainable, community-driven solutions.

## Introduction

Cardiovascular disease persists as the nation’s leading cause of death and follows entrenched social and structural disadvantage that erodes cardiovascular health (CVH) [1]. Social determinants of health (SDoH), the conditions in the environments where people are born, live, learn, work, age, and worship, are among the most powerful drivers of health risk and opportunity across the life course [2]. They influence access to essential resources, exposure to stress, and other fundamental conditions that drive prevention and progression [3]. Although awareness of the connection between SDoH and CVH is growing, the translation of this knowledge into clinical, research, and policy practice remains inconsistent [2]. Tools such as the Accountable Health Communities screening instrument help identify social risks, yet measurement gaps and siloed implementation impede integration into care pathways and scaling [4]. Without clear strategies and actionable solutions, current approaches risk reinforcing fragmented systems and perpetuating CVH disparities. Achieving health equity requires moving beyond documenting CVH disparities to restructuring systems through implementation-focused, community-engaged strategies [5]. This review synthesizes evidence on how SDoH shape cardiovascular health, as defined by the American Heart Association Life’s Essential 8 (LE8)[6], examines each core domain, highlights promising interventions, and identifies gaps to guide equity-driven research, clinical innovation, and policy.

## Defining Social Determinants of Health

Public health efforts to advance CVH equity increasingly rely on frameworks to organize and define the social conditions that shape risk. One of the most widely used efforts, Healthy People 2030, developed by the U.S. Department of Health and Human Services, outlines five key domains of SDoH: economic stability, education access and quality, healthcare access and quality, neighborhood and built environment, and social and community context [7]. While useful for organizing and tracking SDoH, it does not clearly distinguish between structural determinants—upstream policies, institutional practices, and power dynamics shaping resource distribution—and the social conditions they produce, such as food insecurity, unstable housing, under-resourced schools, and limited access to care, which directly influence cardiovascular risk and resilience [2]. Despite these limitations, the Healthy People 2030 framework provides measurable goals and shared priorities that help align clinical, research, and policy efforts.

## Social Determinants of Health and Cardiovascular Health

The connection between social conditions and cardiovascular disease is longstanding, but our understanding of its biological consequences continues to evolve. The weathering hypothesis first suggested that prolonged exposure to racism, economic hardship, and chronic social adversity accelerates health deterioration [8–11]. More recent models, such as the biology of adversity, demonstrate that chronic stress alters immune function, gene expression, and cellular aging, contributing to inflammation, endothelial dysfunction, and atherosclerosis [12, 13]. These physiological changes are compounded by behavioral constraints such as limited access to healthy foods, safe places for activity, and preventive care, which drive the onset of hypertension, obesity, diabetes, and other cardiometabolic conditions [2, 12, 14]. Social disadvantages often cluster, doubling all-cause mortality risk and reinforcing CVH disparities [15].

The American Heart Association LE8 framework captures four key behaviors (diet, physical activity, nicotine exposure, sleep) and four health factors (body mass index [BMI], blood pressure, blood glucose, cholesterol), all shaped by these same social and structural forces [6]. Fewer than one in five U.S. adults achieve high cardiovascular health, characterized by higher composite LE8 scores across behaviors and factors, with LE8 scores consistently lower among racially and ethnically minoritized groups and those with lower socioeconomic status [16, 17]. These inequities persist beyond individual behaviors and health factors, underscoring the independent role of SDoH. [18, 19] While LE8 enables standardized monitoring, translating recognition of social risk into scalable interventions requires domain-specific strategies.

## Economic Stability

### Overall Cardiovascular Health

Economic stability reflects the ability to secure essential health resources including income and financial security (i.e., expenses, debt) alongside employment conditions and work environment (i.e., worker protections, paid sick leave, childcare services) [20]. These factors strongly influence the prevention and management of cardiovascular disease. Higher socioeconomic status—measured by education, income, employment, and insurance—is linked to better LE8 scores, though associations vary across racial, ethnic, and sex groups [21, 22]. The relationship is strongest among non-Hispanic White adults, weaker among Black, Hispanic, and Asian adults, and, in some cases, more pronounced for women [23]. National surveillance data show that among the lowest-income adults, predicted 10-year atherosclerotic cardiovascular disease risk has not improved over three decades despite overall national gains [24].

### LE8 Health Behaviors

Economic strain limits access to healthy foods, safe spaces for physical activity, adequate sleep, and resources for smoking cessation. Food insecurity, in particular, is linked to suboptimal diet quality, less consistent physical activity, disrupted sleep patterns, and low LE8 scores [25–27]. Even participants in safety net programs such as Supplemental Nutrition Assistance Program (SNAP) have disproportionately suboptimal cardiovascular health [28]. Interventions show that economic supports can produce meaningful change. In a randomized controlled trial (RCT) in an urban safety net clinic (*N* = 3,881 patients), weekly fruit and vegetable incentives plus nutrition education improved food security, diet quality, blood pressure, BMI, and HbA1c [29]. A simulation study projected that scaling produce prescriptions to food-insecure adults with diabetes nationwide could prevent nearly 300,000 cardiovascular events and generate $44 billion in productivity and health-related savings [30]. Another RCT found that monthly cash benefits for low-income residents (*N* = 2,880) reduced emergency department visits, especially for behavioral health, and increased access to outpatient subspecialty care [31].

### LE8 Health Factors

Economic disadvantage is linked to worse LE8 health factors for blood pressure, blood glucose, cholesterol, and BMI. A dose–response relationship exists between family income and cardiovascular outcomes [32]. In the ARTEMIS trial, a multicenter study of post–myocardial infarction patients (*N* = 9,590), one year of copayment vouchers for antiplatelet therapy improved adherence and reduced major adverse cardiovascular events, with the greatest benefits among frequent users [33]. Follow-up analyses showed that support only at the point of hospital discharge, such as a one-time voucher or counseling session, did not sustain long-term persistence with P2Y12 inhibitor therapy, underscoring the limits of one-time interventions [34]. Sex differences also emerged, with women less likely than men to redeem or adhere to P2Y12 inhibitor therapy after myocardial infarction, raising concerns about equitable access [34]. The FIReWoRk trial, a multicenter study of adults with obesity in low-income neighborhoods (*N* = 668), found that pairing a commercial weight loss program with financial incentives improved short-term weight loss, though benefits diminished after incentives ended [35].

### Considerations For Economic Stability Interventions

Sustaining CVH gains requires moving from short-term assistance toward integrated, long-term supports. The emerging Food Is Medicine framework, advanced in an American Heart Association Presidential Advisory and Healthcare by Food Initiative, emphasizes embedding produce prescriptions and medically tailored meals into clinical care to prevent and manage diet-related disease [36]. Priority strategies include making SNAP and produce prescriptions permanent, equity-centered models, leveraging Medicaid waivers for tailored meals, and implementing cost-sharing vouchers for essential medications. Policy actions to strengthen income security, lower out-of-pocket healthcare costs, and improve workforce protections can address root inequities. Aligning healthcare, public health, and policy will ensure resources are consistently accessible, affordable, and delivered with dignity [37].

## Education Access and Quality

### Overall Cardiovascular Health

Educational attainment is a powerful predictor of cardiovascular health [38]. Health literacy, the ability to access, understand, evaluate, and use health information to make informed health decisions, is a key pathway linking education to cardiovascular outcomes [39]. Adults without a high school diploma have substantially greater lifetime cardiovascular risk and more years lived with disease compared to college graduates [40, 41]. These disparities persist after adjusting for income, insurance, and early-life socioeconomic status, underscoring education’s independent influence [40, 42]. Yet these benefits remain unequal, with Black adults facing higher cardiovascular risk than equally educated White peers, reflecting the persistent effects of structural racism and cumulative disadvantage [40, 42].

### LE8 Health Behaviors

Educational attainment shapes CVH behaviors, including diet, physical activity, smoking, and sleep. A quasi-experimental study using state-level schooling laws demonstrated that additional years of education were causally linked to lower rates of cardiovascular disease, smoking, and depression [43]. The SAFE HEART intervention, which delivered culturally tailored cardiovascular education via newsletters and webinars, improved CVH literacy among women of reproductive age (*N* = 228), especially in knowledge areas related to diet, physical activity, and medication management [44]. A single-blind RCT in 60 hypertensive patients found that a four-week blended program of face-to-face and online instruction improved diet, medication adherence, and physical activity compared to traditional education [45].

### LE8 Health Factors

Educational attainment is linked to CVH factors, including blood pressure, blood glucose, cholesterol, BMI, and weight status. Among young adults, college graduates are 3.5 to 5 times more likely to meet ideal CVH criteria compared to those without a high school diploma, even after adjusting for race, income, and early-life socioeconomic status [41]. In nationally representative data from over 43,000 adults, both low income and low educational attainment were associated with elevated cardiometabolic risk and premature mortality [46]. A 24-month cluster-RCT in Louisiana primary care clinics (*N* = 803) tested a culturally adapted, health literacy-directed lifestyle program, with participants achieving an average 4.9% weight loss versus 0.44% in usual care [47].

### Considerations For Education Access and Quality Interventions

Improving CVH through education requires building knowledge and enabling its obtainment. Strategies include embedding health literacy instruction across K–12 curricula, especially in underserved communities; adopting universal precautions in healthcare communication; and expanding access to community-based programs delivered by trusted messengers such as faith leaders, barbers, and community health workers [42]. Healthcare organizations can also become health-literate institutions[48], by prioritizing clear communication, simplified navigation, and patient empowerment. Expanding therapeutic patient education may strengthen prevention and self-management[49], but these efforts must be paired with policies addressing economic instability, since the benefits of education are maximized when financial barriers are reduced.

## Health Care Access and Quality

### Overall Cardiovascular Health 

Reliable access to affordable, high-quality care is essential for preventing, detecting, and managing cardiovascular disease. Primary care clinical practice delivers the majority of LE8 screening, counseling, and treatment among adults making it a critical point of engagement for improving CVH and reducing disparities [50]. Despite its importance, access to primary care and preventive cardiovascular services is inconsistent, leaving many adults without timely screening or treatment. During the COVID-19 pandemic, routine cardiovascular screenings declined sharply among low-income adults, especially in non–Medicaid expansion states [51]. These adults were nearly twice as likely to be uninsured, had less access to routine care, and often delayed care due to cost. Even with insurance, monitoring and treatment gaps persisted, suggesting that coverage alone may be insufficient to improve cardiovascular outcomes [52]. Evidence from a large health system shows adults with better CVH had substantially lower healthcare utilization and costs, highlighting the potential for prevention-focused strategies to reduce both disease burden and expenditures [53]. Digital tools such as telehealth, wearable devices, and mobile health applications can extend preventive and chronic care [54], but disparities in digital literacy, broadband, and affordability must be addressed to ensure these innovations narrow rather than widen gaps [55, 56].

### LE8 Health Behaviors

In a study of Mexican origin Latinos in East Los Angeles and Boyle Heights (*N* = 464), having a usual source of care predicted timely screening, counseling, and healthier behaviors such as eating more fruits and vegetables, limiting sugary drinks, and regular physical activity more strongly than insurance status. Insurance alone showed limited associations, underscoring the need to pair coverage with access to regular, culturally responsive care [57]. Access to responsive, community-centered healthcare can facilitate improvements across multiple LE8 behaviors, including diet, physical activity, smoking cessation, and sleep, though evidence for the latter remains limited. A cluster-RCT conducted in Los Angeles barbershops among Black men (*N* = 319) with uncontrolled hypertension showed that trusted community venues can strengthen adherence to dietary guidance, physical activity, and smoking cessation when paired with pharmacist-led care, while yielding notable blood pressure improvements compared to standard referrals [58].

### LE8 Health Factors

Improved access to care is consistently linked with better CVH factors. Medicaid expansion has enhanced outpatient management of hypertension, diabetes, and cholesterol, though disparities remain [59, 60]. An RCT showed that Medicaid coverage reduced systolic blood pressure among adults (*N* = 12,134) who previously had minimal engagement with the healthcare system [61]. Analysis of data from the National Health Interview Survey found adults residing in rural areas had higher rates of hypertension, hyperlipidemia, obesity, diabetes, and coronary heart disease than adults in urban areas, especially among those ages 20–39. These gaps were largely explained by poverty, lower education, and food insecurity [62]. Embedding bilingual community health workers into federally qualified health centers in an RCT in New York City, improved blood pressure control among South Asian adults (*N* = 303) through education, navigation, and follow-up [63]. A 12-week randomized trial of adults with elevated blood pressure also found a telehealth lifestyle program, with or without weekly dietitian-led counseling, reduced 24-h systolic blood pressure and improved sleep, blood pressure control, physical activity, and participants’ satisfaction with the intervention [64].

### Considerations for Health Care Access and Quality Interventions

Expanding equitable cardiovascular care will require coordinated efforts that strengthen both clinical and community-based delivery. Task shifting to and integration of nurses, pharmacists, and community health workers within collaborative care teams, can improve efficiency when supported by sustainable reimbursement structures such as Medicaid [65, 66]. Community-based digital strategies also show promise. The FAITH! Trial, a cluster-RCT intervention in African American churches (*N* = 16, 85 participants), used a co-designed mobile health app to deliver tailored education and achieved improvements in diet, physical activity, and overall CVH scores [67]. Black Impact, a 24-week intervention focused on physical activity and health education, addressed social needs through community health workers, ensured access to a primary care provider, and improved CVH in 70 African American men with suboptimal cardiovascular health [68, 69]. There is limited research on how healthcare access influences CVH behaviors such as diet, physical activity, smoking cessation, and sleep. Future studies should evaluate these pathways to identify effective strategies for translating improved access into sustained lifestyle changes and better cardiovascular outcomes.

## Neighborhood and Built Environment

### Overall Cardiovascular Health 

Neighborhoods influence CVH through access to safe housing, transportation, healthy foods, greenspace, recreational spaces, and healthcare, as well as exposure to environmental stressors such as crime and pollution [70, 71]. Four housing dimensions—stability, quality and safety, affordability and accessibility, and neighborhood environment—directly shape cardiovascular health, with housing insecurity, adverse housing quality, residential segregation, and disadvantaged neighborhood conditions linked to higher rates of cardiovascular disease, hypertension, obesity, and diabetes, particularly among minoritized and low-income populations [71, 72]. Structural tools such as the Area Deprivation Index, redlining maps, and EMR-linked neighborhood data help quantify risk and reveal persistent associations between historical disinvestment and current disparities [71]. Systematic reviews and policy guidance highlight that walkable neighborhoods, mixed land use, traffic safety, and park access improve cardiovascular profiles through increased physical activity, improved diet, and reduced stress [73–75].

### LE8 Health Behaviors

Neighborhoods with supportive infrastructure promote healthier behaviors. A systematic review found that higher walkability, residential density, and recreational facility access were associated with greater physical activity, while fast-food density and limited supermarket access were linked to unhealthy diets and reduced physical activity [74]. A multi-method study using GPS mapping, ecological momentary assessment, and street-view audits found that lower perceived safety and higher neighborhood deprivation predicted reduced activity and higher obesity, especially in low-income and racially minoritized groups [76]. In Hamtramck, Michigan, a community-led alley activation project used low-cost infrastructure improvements to enhance safety and appeal; residents reported more willingness to walk, greater pedestrian activity, and stronger social connections supporting physical activity and lower stress [77]. In the Project Viva cohort of midlife women, greater neighborhood tree canopy coverage was linked to better diet, higher physical activity, healthier sleep, lower tobacco exposure, and more favorable BMI and blood pressure [78].

### LE8 Health Factors

Neighborhood context is closely linked to measurable CVH outcomes. Analysis of a national cohort of more than 770,000 Veterans with atherosclerotic cardiovascular disease found that suboptimal built environment features, such as derelict buildings and exposed wiring were significantly associated with increased major adverse cardiovascular event risk, with patterns differing by social deprivation and urban versus rural location [79]. A spatial epidemiologic study of premature cardiovascular disease mortality in Atlanta found that Black adults accounted for 85% of deaths despite representing just over half of the population aged 35–64, with a mortality rate of 15.6 per 1,000 compared with 6.0 per 1,000 among White adults [80]. Initial associations between limited food access, low walkability, and higher mortality disappeared after adjusting for racial composition and poverty, underscoring the influence of structural racism [80].

### Considerations for Neighborhood and Built Environment Interventions

Improving neighborhood conditions requires both structural reforms and locally driven solutions. Policy action to reverse inequities created by historical practices such as redlining is essential, including investments in affordable, stable housing; zoning reforms to support mixed land use; expanded public transit; and equitable distribution of parks, green space, and safe recreational infrastructure [72, 75]. Integrating neighborhood risk indicators, such as the Area Deprivation Index, into healthcare systems can help clinicians identify environmental barriers to CVH and connect patients to resources. Community level strategies such as walk audits, greening initiatives, mobile health units, and quick-build pedestrian safety improvements can build social cohesion, enhance perceptions of safety, and promote daily physical activity. These approaches are most effective when paired with upstream policy change to sustain benefits. Evidence from Philadelphia shows that relatively low-cost environmental interventions can improve health: vacant lot greening reduced depression and improved mental health [81], while abandoned housing remediation lowered gun violence [82].

## Social and Community Context

### Overall Cardiovascular Health 

Social support, isolation, and loneliness are central to the social and community context domain, influencing CVH through behavioral, psychosocial, and biological pathways [83]. Lower social support and higher social isolation or loneliness are consistently linked with elevated cardiovascular risk. In postmenopausal women, greater social isolation and loneliness predicted higher incidence of cardiovascular disease [84], while the Multi-Ethnic Study of Atherosclerosis found stronger emotional and social support reduced cardiovascular events and chronic stress increased risk [85]. One cross-sectional study of African American adults within the Jackson Heart Study (*N* = 2,967) showed that religiosity and spirituality were associated with greater odds of achieving ideal CVH behaviors and factors, underscoring protective sociocultural factors in African American communities [86]. Prospective data further indicate that strong perceived social support slows disease progression and lowers all-cause mortality among patients with established cardiovascular disease, with some studies suggesting stronger effects in women than in men [87]. Another analysis of the Jackson Heart Study of over 4,000 Black adults showed that chronic stress and major life events undermined ideal CVH behaviors and factors, particularly smoking cessation and glucose control [88].

### LE8 Health Behaviors

Perceived social support strongly shapes lifestyle behaviors that influence cardiovascular health. A cross-sectional study of patients with heart failure, hypertension, coronary artery disease, and chronic arrhythmias, examined the relationship between perceived social support and key lifestyle behaviors, focusing on support from family, friends, and significant others [89]. Participants with higher perceived social support reported greater adherence to medical appointments, healthier dietary patterns, and lower tobacco and alcohol use. A multi-center observational study of patients with heart failure, coronary artery disease, and other cardiovascular conditions found similar links, with greater support tied to improved quality of life, consistent healthcare engagement, and reduced substance use [90]. Together, these findings indicate that enhancing social support must be coupled with strategies to reduce stressors that erode healthy lifestyle choices.

### LE8 Health Factors

Social support has measurable associations with CVH factors. In a national cohort of nonelderly adults, higher perceived social support predicted better LE8 scores, including lower BMI, healthier blood pressure, and better glucose control, even after adjustment for socioeconomic and clinical factors [91]. A four-month RCT in Philadelphia found that remote blood pressure monitoring achieved similar control rates, with or without a support partner, suggesting low-intensity support may be insufficient for sustained change [92]. By contrast, a ten-month “microclinics” program in rural Appalachia, built around friend and family networks, produced sustained improvements in weight, waist circumference, blood pressure, and HbA1c [93]. The Black Impact pilot trial among Black men further underscored the role of social support, with participants highlighting peer “brotherhood” and health coach encouragement as key drivers of participation, accountability, and improvement across CVH behaviors [94].

### Considerations for Social and Community Interventions

Strengthening social and community connections is an underused pathway to improve cardiovascular health. Approaches that foster cohesion, trust, and supportive networks can counter isolation, loneliness, and chronic stress. Examples include peer support programs in clinical care, community health worker outreach, and neighborhood initiatives that enhance safety, public spaces, and engagement opportunities [95, 96]. Healthcare systems can screen for isolation and connect patients to social prescribing programs, faith-based organizations, or community centers. Partnerships with local organizations can extend reach, especially in underserved communities, though more cardiovascular-specific trials are needed to confirm impact. Table 1.

**Table 1:**
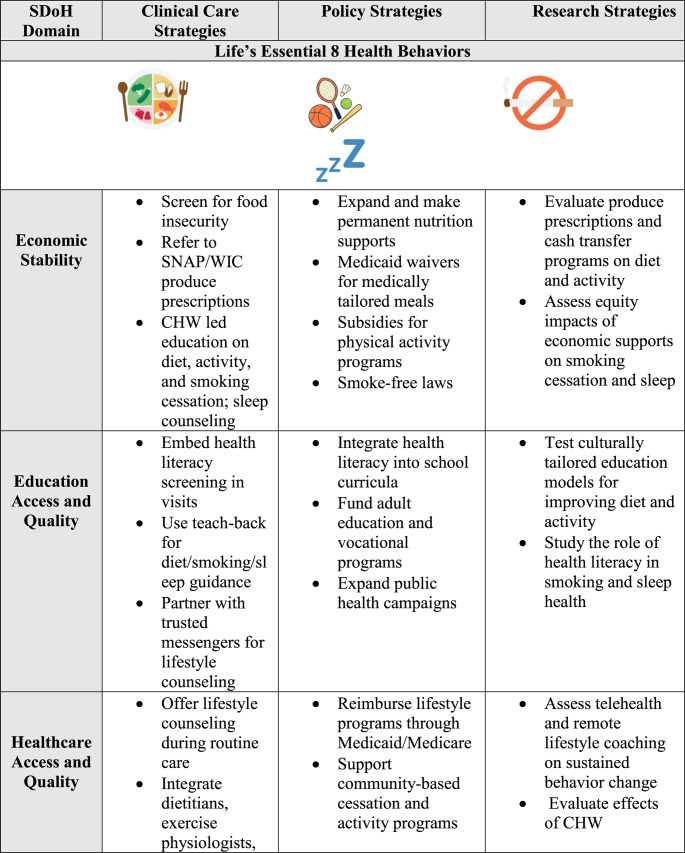

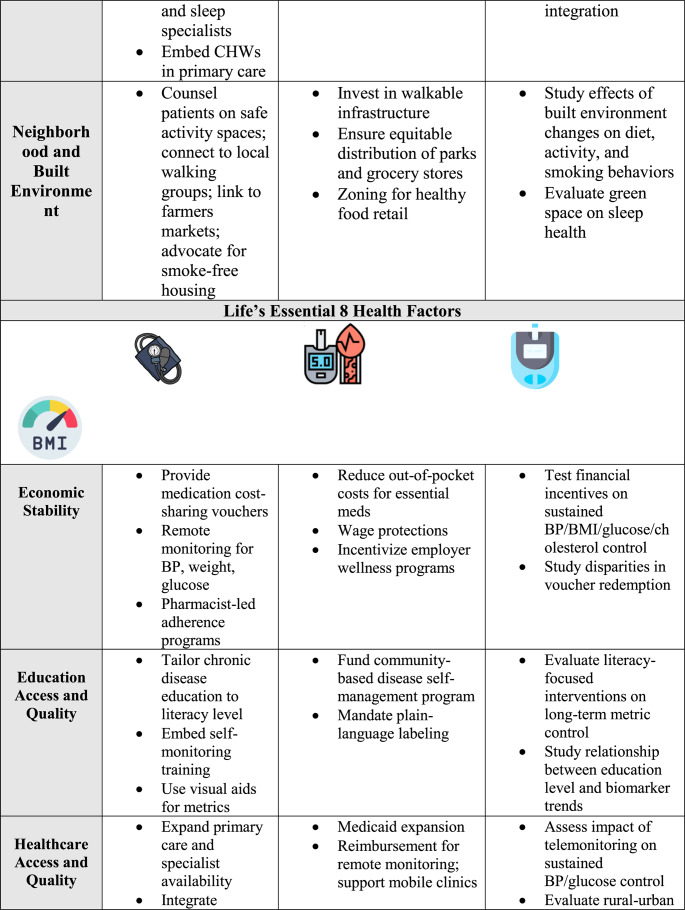

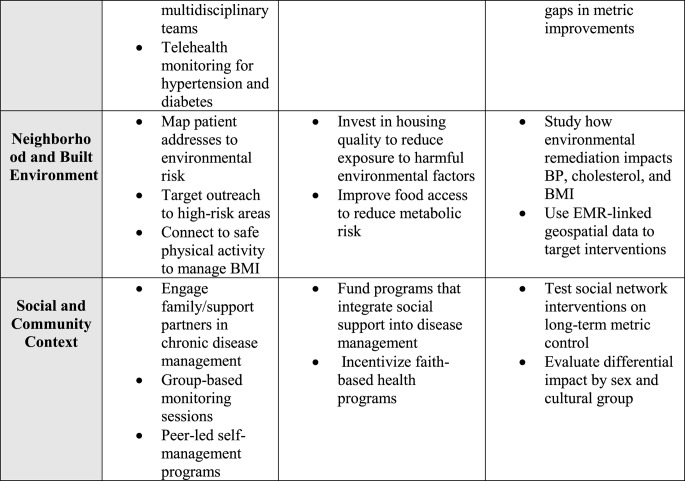
Social determinants of health (SDoH) strategies addressing life’s essential 8 behaviors and factors

*CHW* Community health worker, *SNAP *Supplemental Nutrition Assistance Program, *BMI *Body Mass Index, *EMR *Electronic Medical Record

## Key Considerations for Practice, Policy, and Research

**Fig. 1:**
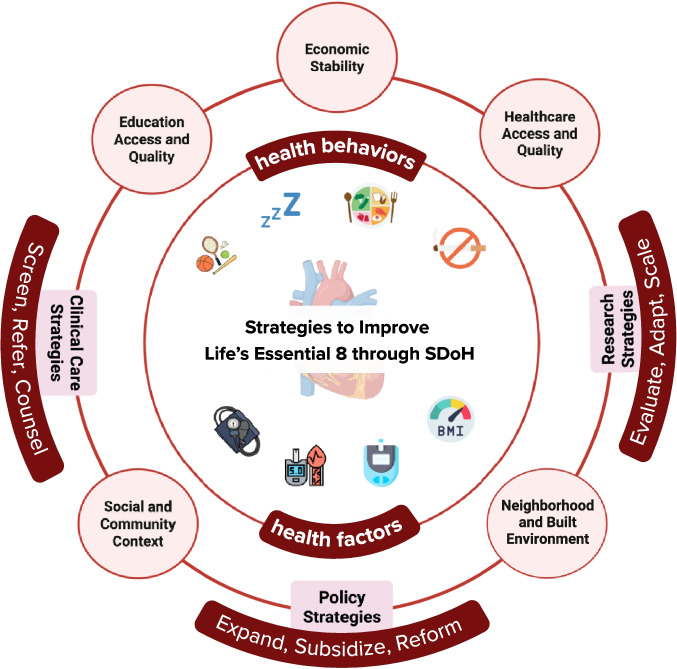
Conceptual model showing how strategies across clinical care, policy, and research can improve the American Heart Association Life’s Essential 8. The Healthy People 2030 social determinants of health domains (economic stability, education, healthcare access and quality, neighborhood and built environment, and social and community context) act as levers to influence CVH outcomes through integrated approaches

Improving CVH requires aligning clinical care, policy, and research to address SDoH across all LE8 domains. These factors shape diet, physical activity, nicotine exposure, sleep health, BMI, blood pressure, cholesterol, and blood glucose through interconnected behavioral, physiological, and structural pathways. Figure 1.

## Clinical Practice

Routine assessment of both social risk and LE8 factors should be standard in care, using validated tools such as the Accountable Health Communities screening instrument[4] and integrating results into electronic health records. Screening must be paired with clear referral pathways to community health workers, navigators, or social service agencies to address needs such as food, housing, and transportation. Navigation alone may have limited effect without sufficient intensity or resources; sustained, multi-component approaches are more likely to improve outcomes. CVH literacy should be treated as a vital sign, with culturally tailored, plain-language education from trusted messengers. Community-based delivery models—nurse- or pharmacist-led programs in churches, barbershops, and community centers—and digital tools, such as telehealth-supported lifestyle counseling, can strengthen engagement and improve LE8 scores [97, 98].

## Policy

Structural solutions can directly improve LE8 behaviors and factors. Expanding nutrition supports and produce subsidies can raise diet quality, while zoning reform, safe transportation, and urban greening promote physical activity. Smoke-free policies and cessation programs reduce nicotine exposure, and digital equity policies ensure that effective tools such as telehealth and remote monitoring for blood pressure control are accessible to all. Sustained funding for Federally Qualified Health Centers[66], Medicaid expansion, and reimbursement for community-based prevention models will improve access to preventive care and chronic disease management. Payment models that reward improvement in LE8 factors can incentivize prevention-focused care.

## Research

Future studies should move beyond describing and reciting persistent CVH disparities to testing multilevel interventions that address upstream determinants and improve all LE8 components. Longitudinal research can examine how policies such as wage increases or housing subsidies influence BMI, blood pressure, and diet over time. Implementation science can identify how to adapt evidence-based strategies across diverse settings, and equity-focused trial designs can ensure benefits reach those at highest risk [99]. Greater attention to underrepresented LE8 behaviors and factors, such as sleep health, paired with participatory research approaches will ensure communities are active partners in designing and scaling solutions.

## Conclusions

SDoH are inextricably linked to cardiovascular risk, influencing both the causes and consequences of disease across the life course. To improve cardiovascular outcomes and close equity gaps, it is not enough to simply acknowledge these factors—we must act on them. This requires a collective commitment to redesigning health systems, investing in communities, and aligning research, policy, and clinical care around strategies that meet people where they are.

## Key References

- Brewer LC, Jenkins S, Hayes SN, Kumbamu A, Jones C, Burke LE, Cooper LA, Patten CA. Community-Based, Cluster-Randomized Pilot Trial of a Cardiovascular Mobile Health Intervention: Preliminary Findings of the FAITH! Trial. *Circulation*. 2022;146:175–190. 10.1161/CIRCULATIONAHA.122.059046.
  - ○ Findings from this study suggest that a culturally tailored, community-based mobile health program can improve cardiovascular health behaviors among African American adults.
- Islam NS, Wyatt LC, Ali SH, Zanowiak JM, Mohaimin S, Goldfeld K, Lopez P, Kumar R, Beane S, Thorpe LE, et al. Integrating Community Health Workers into Community-Based Primary Care Practice Settings to Improve Blood Pressure Control Among South Asian Immigrants in New York City: Results from a Randomized Control Trial. *Circulation: Cardiovascular Quality and Outcomes*. 2023;16:e009321. 10.1161/CIRCOUTCOMES.122.009321.
  - ○ Findings from this study suggest that integrating community health workers into primary care improves blood pressure control among South Asian immigrants.
- Joseph JJ, Nolan TS, Williams A, McKoy A, Zhao S, Aboagye-Mensah E, Kluwe B, Odei JB, Brock G, Lavender D, et al. Improving cardiovascular health in black men through a 24-week community-based team lifestyle change intervention: The black impact pilot study. *Am J Prev Cardiol*. 2022;9:100315. 10.1016/j.ajpc.2022.100315.
  - ○ Findings from this study suggest that peer support and team-based lifestyle interventions can improve cardiovascular health scores and reduce social needs among Black men.

## Data Availability

No datasets were generated or analysed during the current study.

## Abbreviations

SDoH: Social Determinants of Health
LE8: Life’s Essential 8
SNAP: Supplemental Nutrition Assistance Program
CVH: Cardiovascular Health
RCT: Randomized Controlled Trial
HbA1c: Hemoglobin A1c
